# Supplementary material for: PKA-RIIβ autophosphorylation modulates PKA activity and seizure phenotypes in mice
Source: Commun Biol. 2021 Mar 1;4:263. doi: 10.1038/s42003-021-01748-4 (PMC7921646; doi:10.1038/s42003-021-01748-4)
Supplement: Supplementary file 2 — Supplementary Information [file 42003_2021_1748_MOESM2_ESM.pdf]

Supplementary Information

PKA-RIIβ autophosphorylation modulates PKA activity and seizure phenotypes in mice

Jingliang Zhang[1,8], Chenyu Zhang[2,8], Xiaoling Chen[1], Bingwei Wang[2], Weining Ma[3], Yang Yang[4,] Ruimao Zheng[2,5,6,7*], Zhuo Huang[1,6,7*]

1. State Key Laboratory of Natural and Biomimetic Drugs, Department of Molecular and Cellular Pharmacology, School of Pharmaceutical Sciences, Peking University Health Science Center, Beijing 100191, China;
2. Department of Anatomy, Histology and Embryology, School of Basic Medical Sciences, Peking University Health Science Center, Beijing 100191, China;
3. Department of Neurology, Shengjing Hospital Affiliated to China Medical University, Shenyang, 110000, China;
4. Department of Medicinal Chemistry and Molecular Pharmacology, Purdue University College of Pharmacy, 575 Stadium Mall Drive, West Lafayette, IN 47907, USA;
5. Neuroscience Research Institute, Peking University, Beijing, China;
6. Key Laboratory for Neuroscience, Ministry of Education, Beijing 100191, China;
7. Key Laboratory for Neuroscience of National Health Commission, Beijing, China;
8. These authors contributed equally: Jingliang Zhan, Chenyu Zhang.
* These authors jointly supervised this work. Correspondence:

Zhuo Huang, Ph.D.
Department of Molecular and Cellular Pharmacology, Peking University Health Science Center, 38 Xue Yuan Road, Beijing 100191, China
E-mail: huangz@hsc.pku.edu.cn;

Ruimao Zheng, Ph.D.
Department of Anatomy, Histology and Embryology, School of Basic Medical Sciences, Peking University Health Science Center, 38 Xue Yuan Road, Beijing 100191, China
E-mail: rmzheng@pku.edu.cn

This PDF file includes:

Supplementary figures and figure legends: Supplementary Figs. 1-9.
Supplementary tables: Supplementary Tables 1-11.

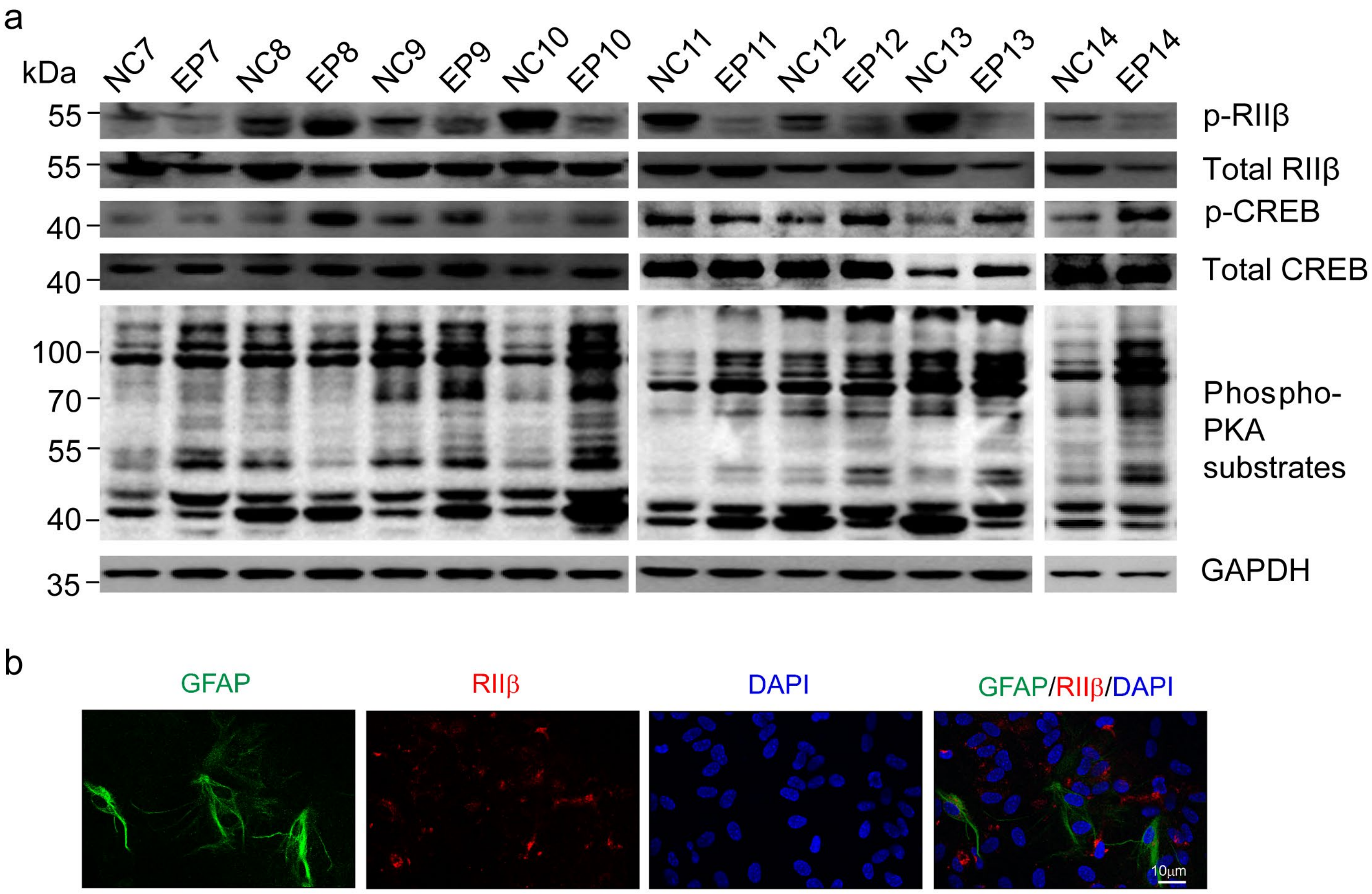

Supplementary Fig. 1 Decreased autophosphorylation of the RIIβ subunit of PKA and increased phosphorylation of CREB and PKA substrates in patients with temporal lobe epilepsy. (related to Fig. 1a-g) **a** Western blot analysis of p-RIIβ, total RIIβ, p-CREB, total CREB, and p-PKA substrates in the epileptic foci and adjacent normal tissue from individuals with TLE. NC: normal control using adjacent normal tissues from a designated epilepsy patient; EP: epileptic focus from a designated epilepsy patient. If the EP was the hippocampus, then the NC was the entorhinal cortex embedded in the anterior temporal lobe; if the EP was the entorhinal cortex in the anterior temporal lobe, then the NC was the hippocampus. **b** Immunostaining of glial fibrillary acidic protein (GFAP, a biomarker of astrocytes, green), RIIβ protein (red), and DAPI (nuclei marker, blue) in primary cultures of glial cells.

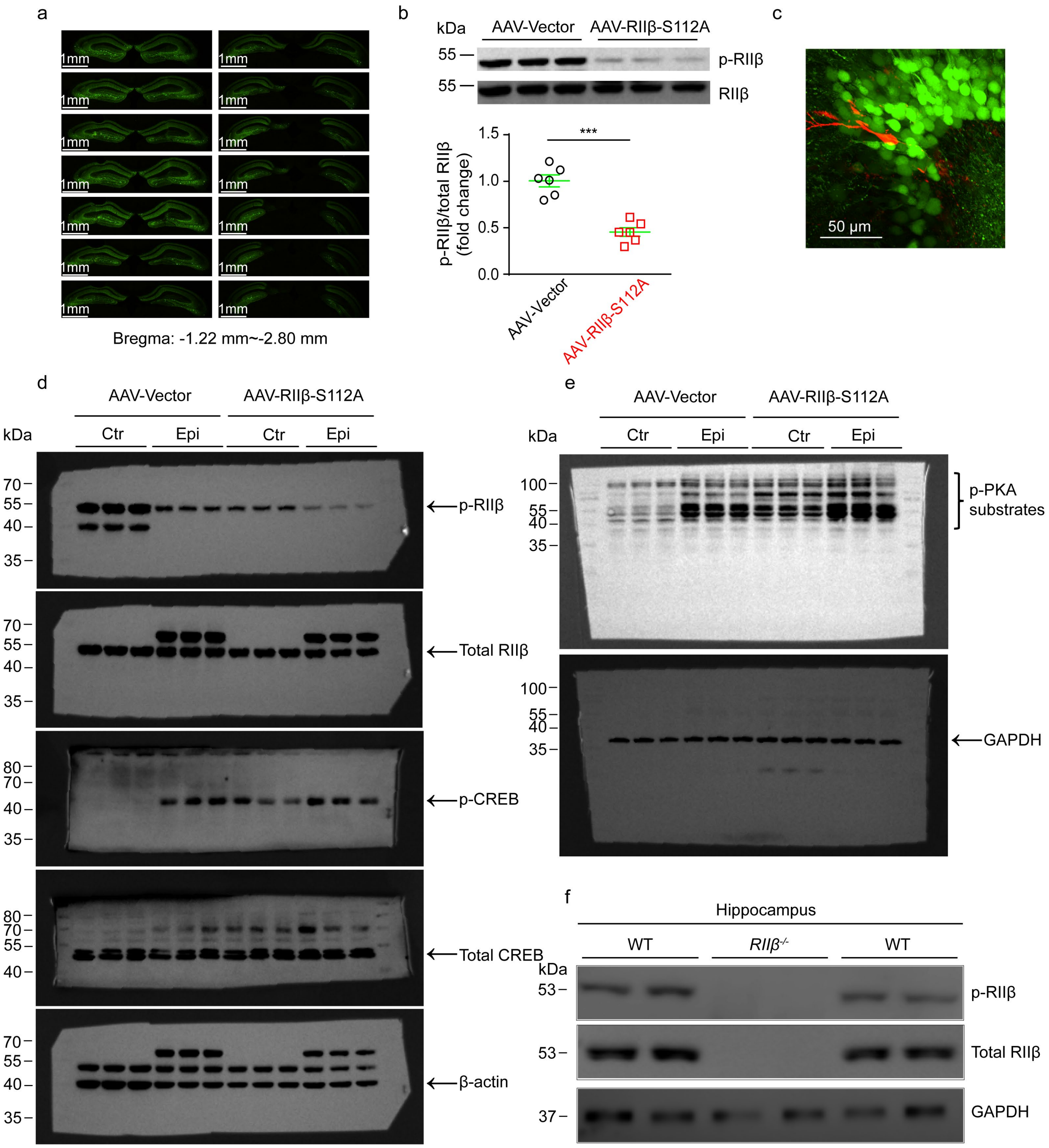

Supplementary Fig. 2 The autophosphorylation of RIIβ was downregulated by AAV-RIIβ-S112A transduction *in vivo*. (related to Fig. 2) **a** Expression of adeno-associated virus in the hippocampal DG area (Bregma: -1.22 mm ~ -2.80 mm). *scale bar*, 1 mm. **b** Western blot analysis showed p-RIIβ and total RIIβ protein levels in the hippocampal DG area of mice in AAV-Vector or AAV-RIIβ-S112A group. n = 6 mice for each group, ***$P < 0.001$, paired two-tailed Student's t-test. Data were represented as mean ± SEM. **c** Mice transfected with AAV were transcardially perfused on day 21 after AAV injection. Scale bar, 50 μm, green indicating AAV-transduced DG granule cells and red indicating one AAV-transduced DG granule cell labeled by neurobiotin. **d-e** uncropped blots for panel (**b**) and **Fig. 2b-c**. **f** Western blot analysis showed p-RIIβ and RIIβ protein signals were absent from the hippocampal lysates of *RIIβ*$^{-/-}$ mice.

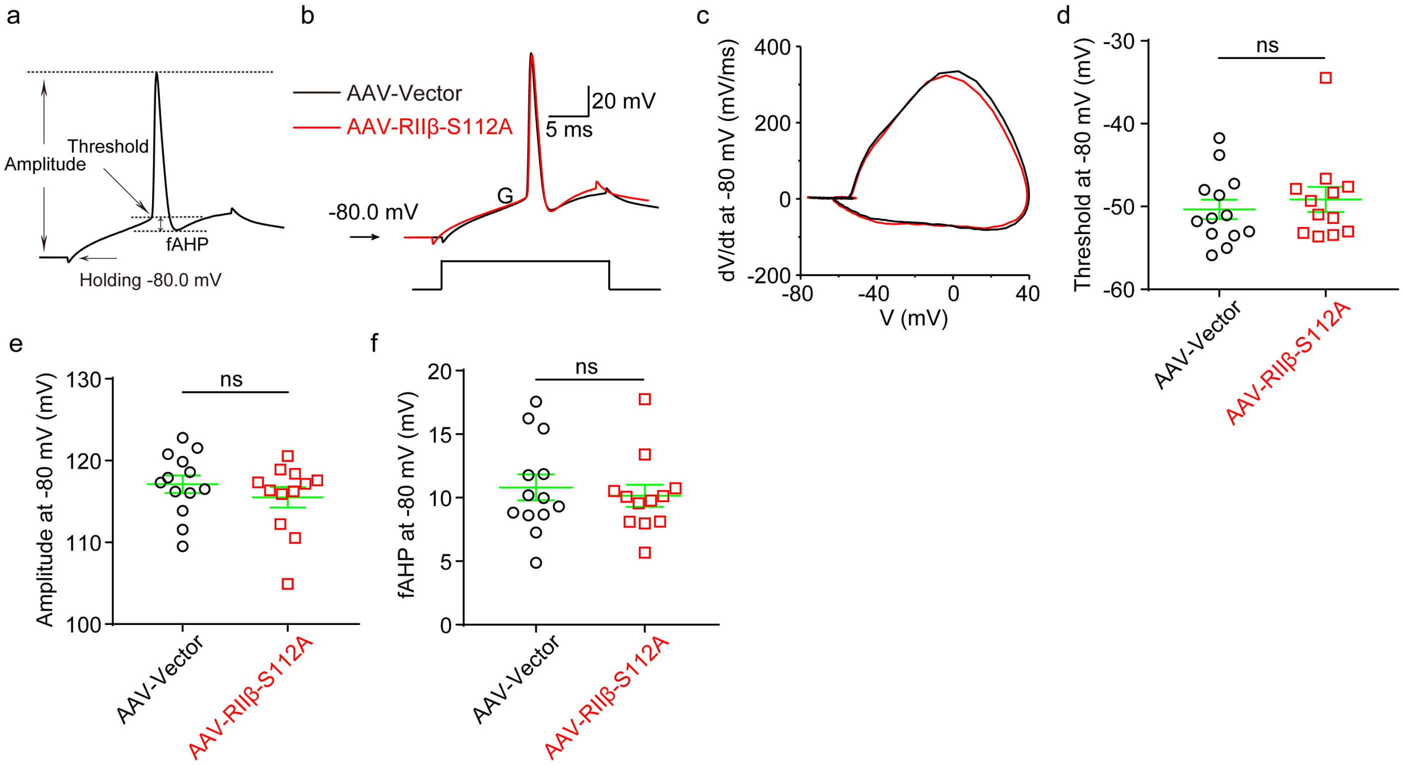

Supplementary Fig. 3 Downregulation of autophosphorylation of RIIβ *in vivo* did not significantly change the AP waveform of DG granule cells when held at a fixed potential of -80 mV in mice. (related to Fig. 5) **a** Plot of a typical action potential showed its various phases as the action potential passes a point on a cell membrane. **b** Typical spikes of DG neurons from mice transfected with AAV-Vector (black) and AAV-RIIβ-S112A (red). **c** Associated phase plane plots. **d-f** Individuals and mean spike threshold, amplitude, and fAHP values. n = 13 neurons for AAV-Vector group and n = 12 neurons for AAV-RIIβ-S112A group, ns: no significance, $P > 0.05$, unpaired two-tailed Student's t-test. Data were represented as mean ± SEM.

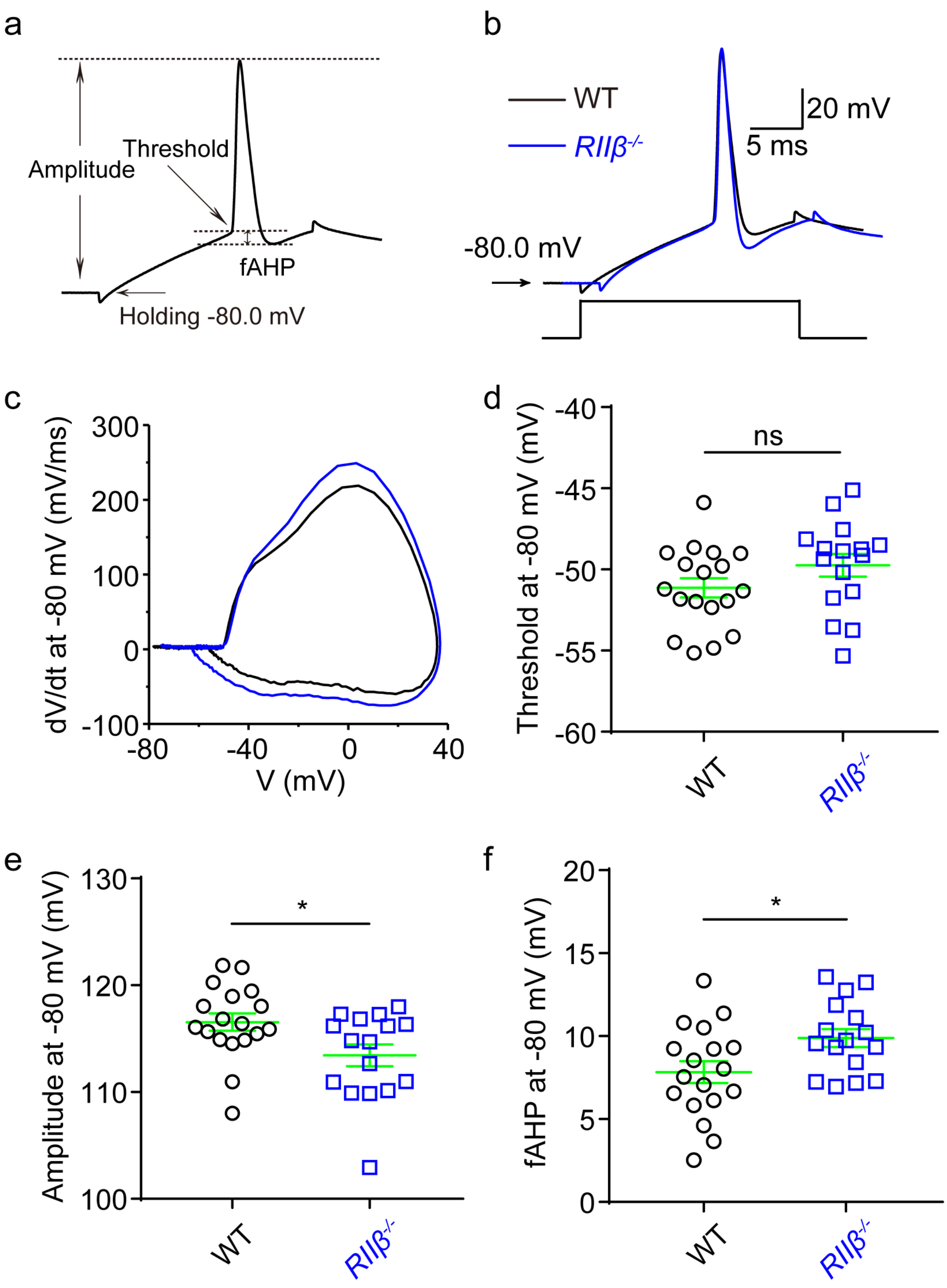

Supplementary Fig. 4 *RIIβ* null mice have a reduced neuronal intrinsic excitability of DG granule cells when held at a fixed potential of -80 mV. (related to Fig. 6) **a** Plot of a typical action potential showed its various phases as the action potential passes a point on a cell membrane. **b** Typical spikes of DG neurons from WT (black) and $RII\beta^{-/-}$ (blue) mice. **c** Associated phase plane plots. **d-f** Individuals and mean spike threshold, amplitude, and fAHP values. n = 18 neurons for WT group and n = 16 neurons for $RII\beta^{-/-}$ group, ns: no significance, $P > 0.05$; $^*P < 0.05$, unpaired two-tailed Student's t-test. Data were represented as mean ± SEM.

Uncropped blots related to Fig. 1a

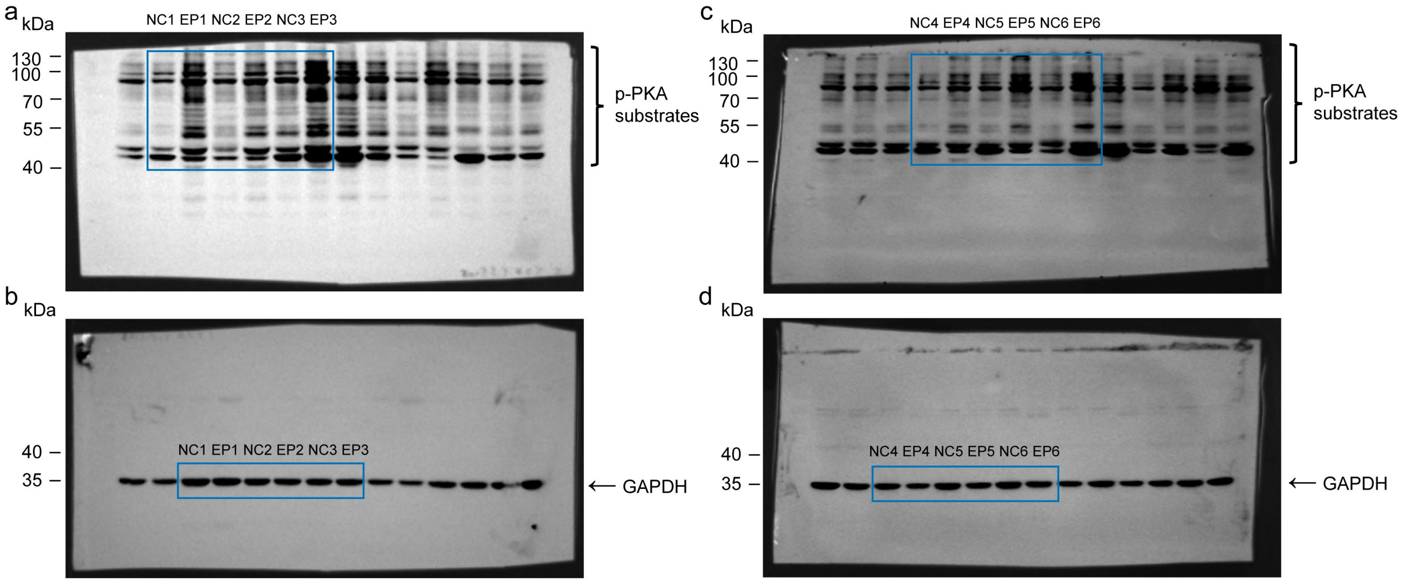

Uncropped blots related to Fig. 1m

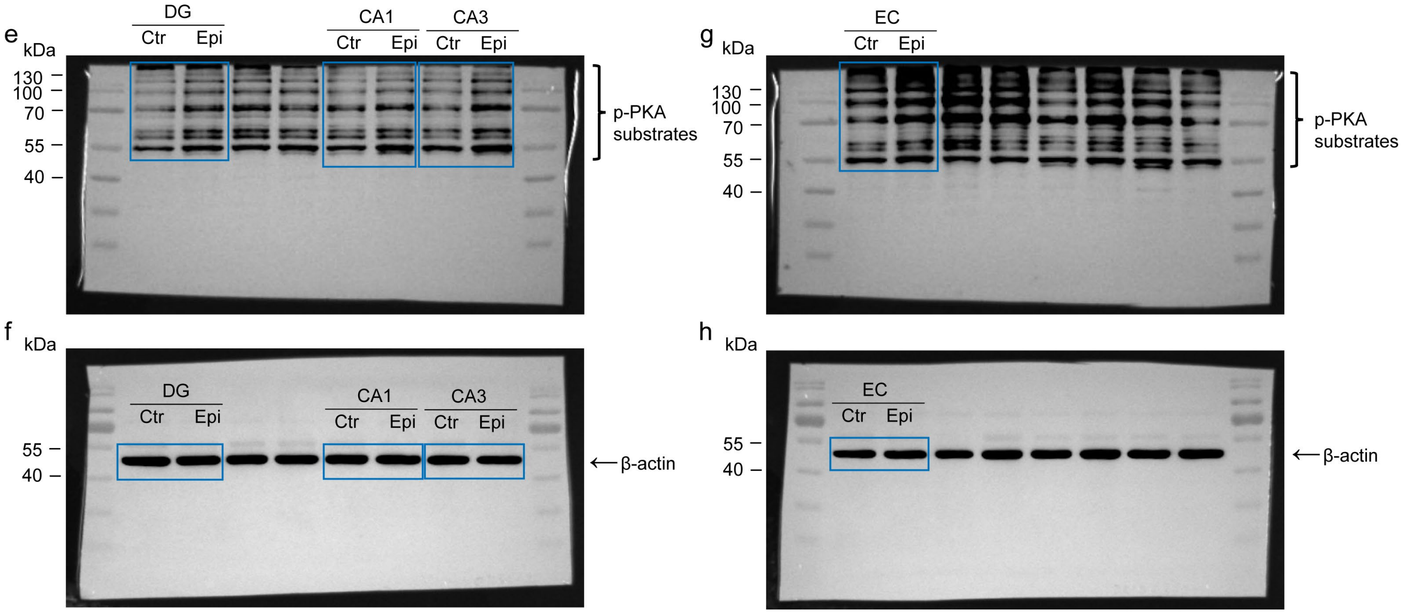

Supplementary Fig. 5 Uncropped blots. (related to Fig. 1) **a-d** uncropped blots for **Fig. 1a**. **e-h** uncropped blots for **Fig. 1m**.

Uncropped blots related to Fig. 1c

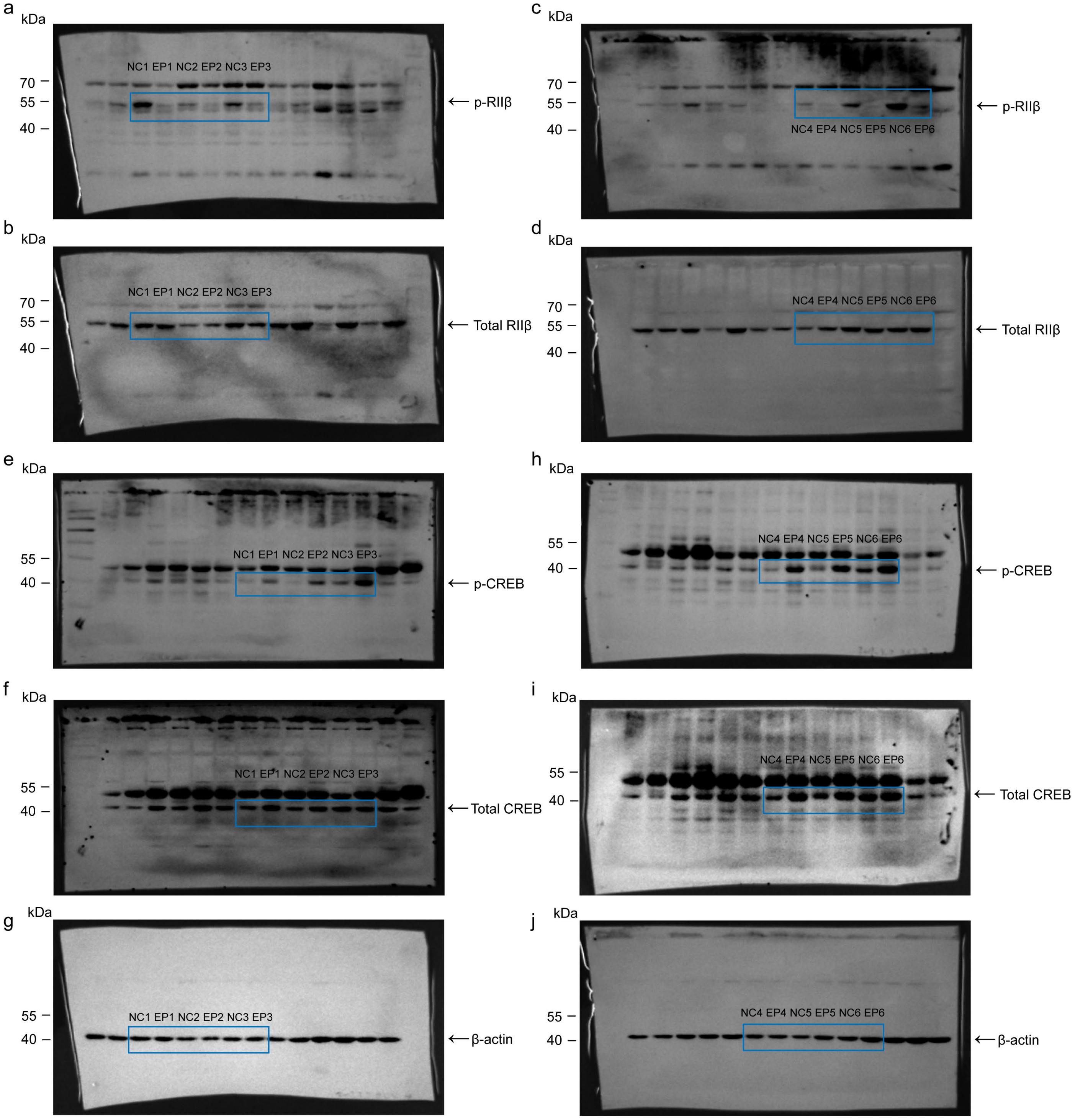

Supplementary Fig. 6 Uncropped blots. (related to Fig. 1) **a-j** uncropped blots for **Fig. 1c**.

Uncropped blots related to Fig. 1h

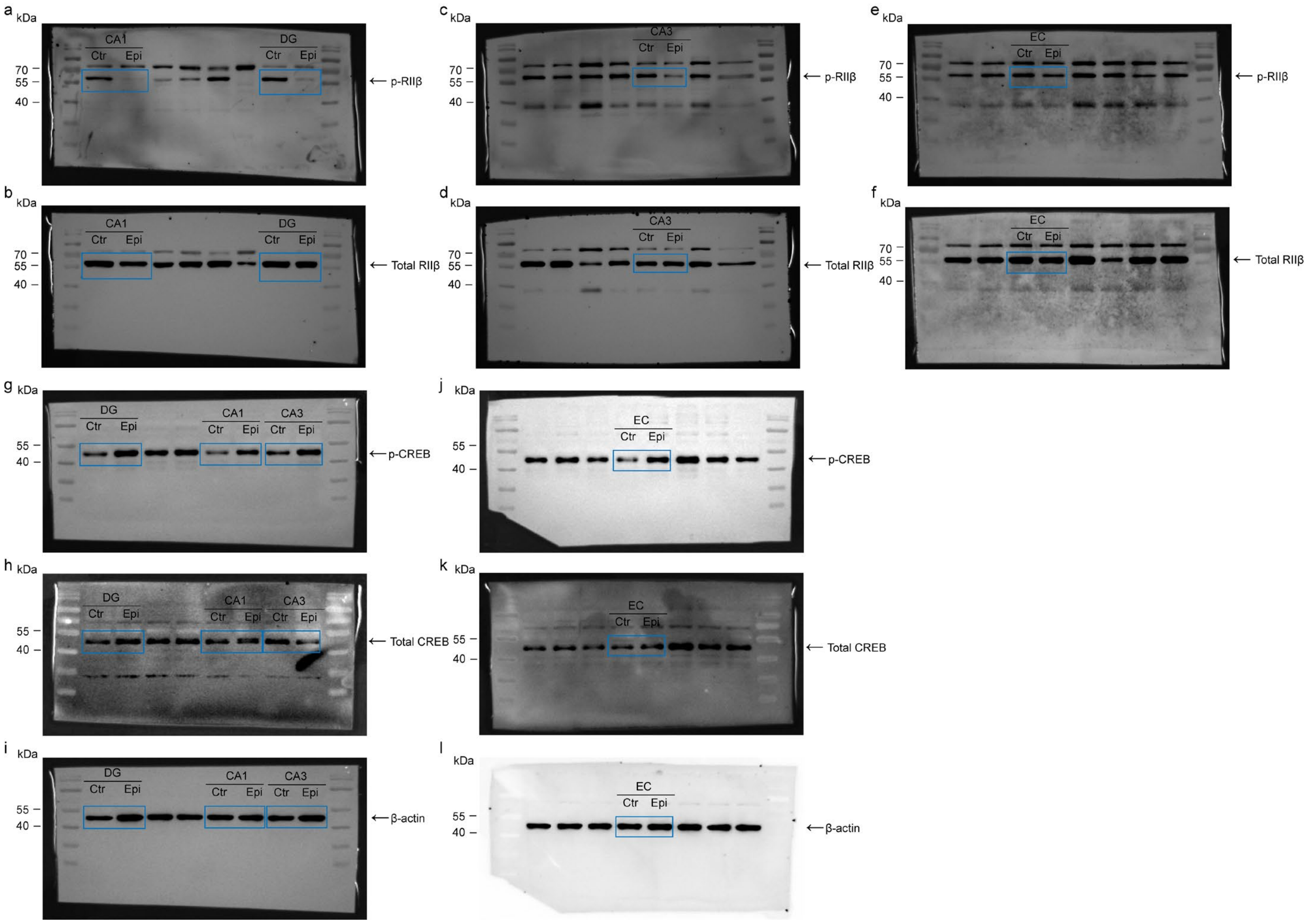

Supplementary Fig. 7 Uncropped blots. (related to Fig. 1) **a-l** uncropped blots for **Fig. 1h**.

Uncropped blots related to Fig. 3a

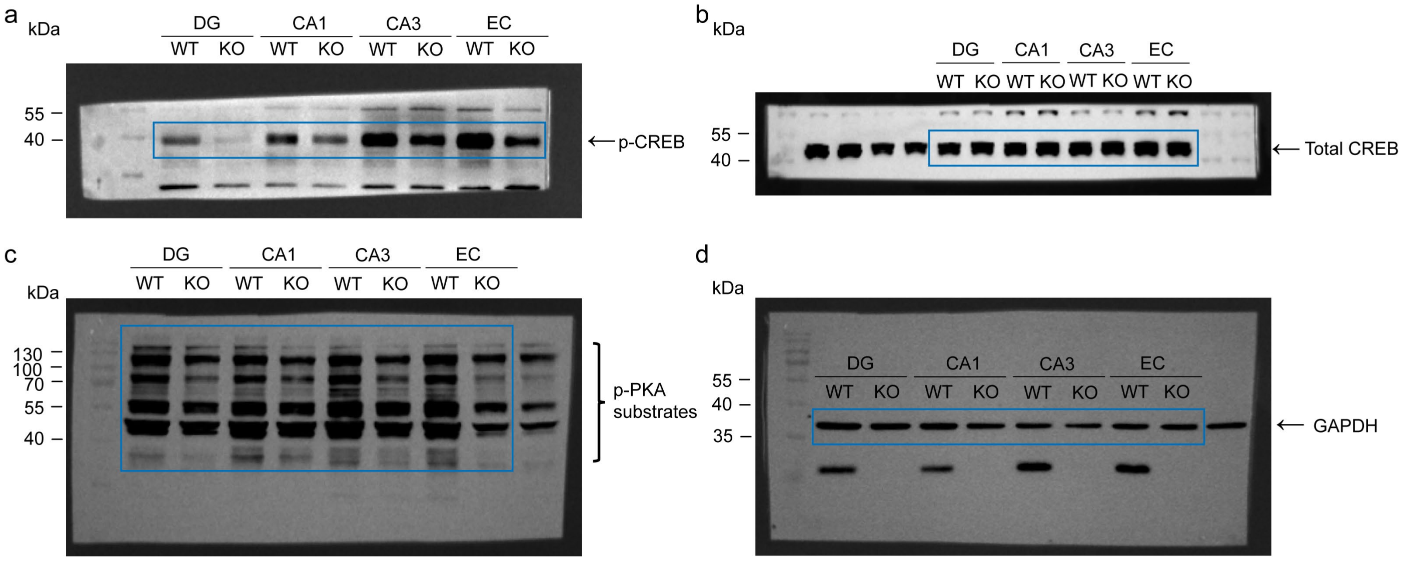

Supplementary Fig. 8 Uncropped blots. (related to Fig. 3) **a-d** uncropped blots for **Fig. 3a**.

Uncropped blots related to Supplementary Fig. 1a

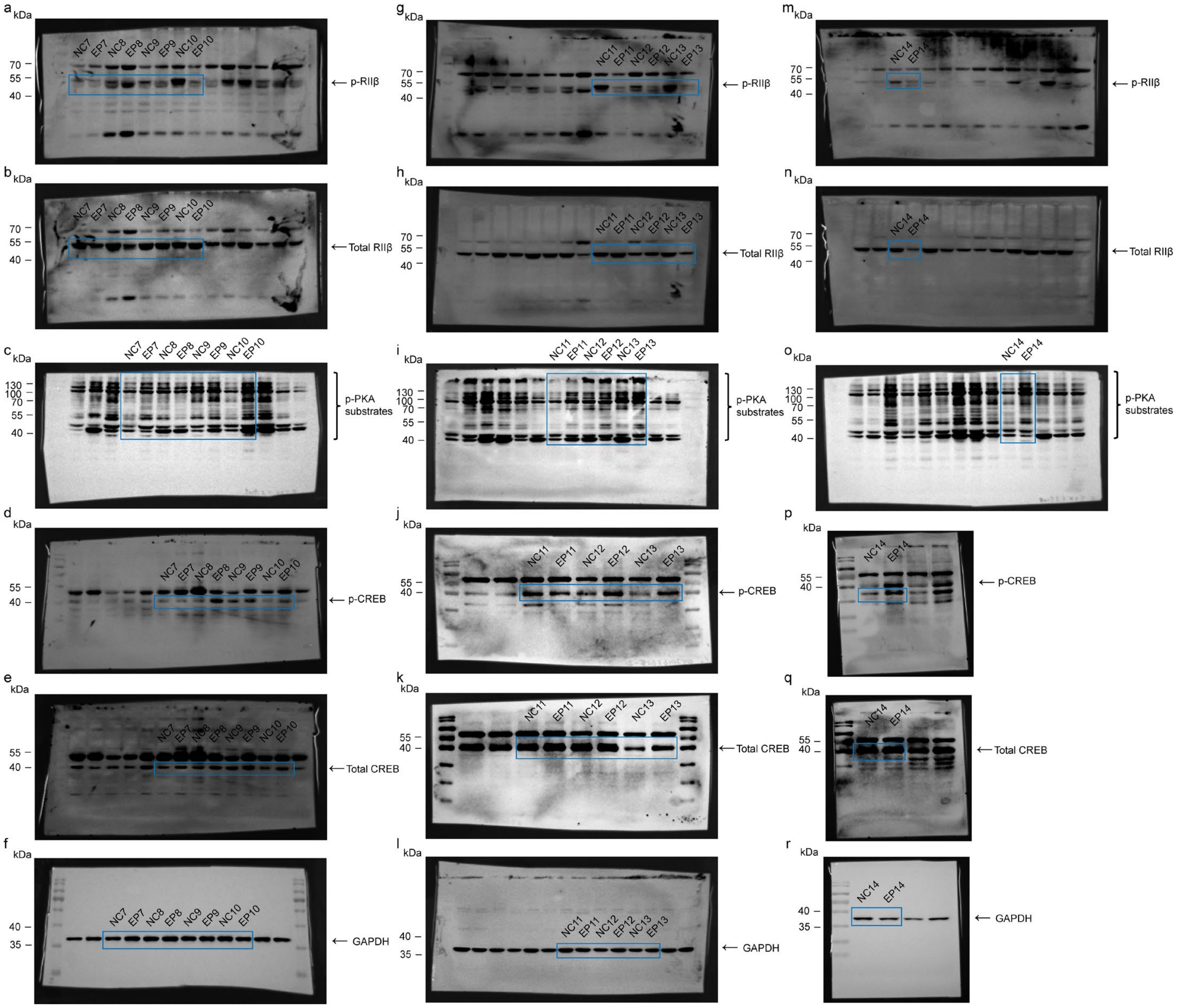

Supplementary Fig. 9 Uncropped blots. (related to Supplementary Fig. 1) **a-r** uncropped blots for **Supplementary Fig. 1a**.

Supplementary Table 1. Demographic and clinical characteristics of selected patients with mesial TLE. (related to Fig. 1a-g and Supplementary Fig. 1a)

Patients* (Number)	Age (Years)	Sex	Time since first unprovoked seizure (Years)	Medication history	MRI examination	Epileptic type#	Type of surgery
1	43	Male	15	Carbamazepine (CBZ)<br>Phenytoin (PHT)	Normal	Medically intractable mesial temporal lobe epilepsy (mTLE)	Anterior temporal lobe and Hippocampus-Amygdala resection
2	30	Male	25	Carbamazepine (CBZ)<br>Phenytoin (PHT)	Normal		
3	44	Male	3	Valproate (VPA)	L temporal-lobe atrophy, signal changes		
4	55	Female	4	--	R hippocampal signal changes		
5	46	Female	40	Valproate (VPA)	R hippocampal atrophy, signal changes		
6	44	Male	10	Carbamazepine (CBZ)<br>Levetiracetam (LEV)<br>Ocazepine (OXC)	R hippocampal atrophy, signal changes		
7	46	Female	10	Valproate (VPA)	Normal		
8	36	Female	15	Valproate (VPA)	Normal		
9	25	Male	11	Valproate (VPA)<br>Levetiracetam (LEV)<br>Ocazepine (OXC)<br>Phenobarbital (PB)	L hippocampal signal changes		
10	44	Female	30	Valproate (VPA)<br>Clonazepam (CZP)	Normal		
11	25	Male	14	Carbamazepine (CBZ)<br>Lamotrigine (LTG)	L hippocampal swellen, signal changes		
12	39	Male	25	Phenytoin (PHT)	Normal		
13	43	Female	4	--	L hippocampal signal changes		
14	4	Male	3	Phenobarbital (PB)<br>Clonazepam (CZP)	L temporal-lobe and hippocampal atrophy, no signal changes		

*All selected patients were seizure-free at the follow up time points after surgery.

#Seizure on set tissues from hippocampus confirmed by scalp and intracranial EEG.

Supplementary Table 2. mIPSC parameters in DG granule cells in the presence of pharmacological blockers. (related to Fig. 4a-c)

mIPSC	n	Frequency (Hz)	Rise 10-90% (ms)	Decay 90-37% (ms)	Half-width (ms)	Peak Amplitude (pA)	Area Under Curve (pAms)
AAV-Vector	17	2.69 ± 0.20	0.60 ± 0.03	8.61 ± 0.43	7.57 ± 0.34	-38.38 ± 1.94	-498.61 ± 19.50
AAV-RIIβ-S112A	18	1.72 ± 0.16***	0.61 ± 0.02	8.37 ± 0.22	7.45 ± 0.19	-37.13 ± 1.72	-481.28 ± 22.32

***$P < 0.001$ compared with AAV-Vector group

Supplementary Table 3. mEPSC parameters in DG granule cells in the presence of pharmacological blockers. (related to Fig. 4d-f)

mEPSC	n	Frequency (Hz)	Rise 10-90% (ms)	Decay 90-37% (ms)	Half-width (ms)	Peak Amplitude (pA)	Area Under Curve (pAms)
AAV-Vector	18	0.67 ± 0.08	1.08 ± 0.09	5.81 ± 0.20	5.23 ± 0.20	-11.26 ± 0.30	-98.91 ± 7.38
AAV-RIIβ-S112A	18	0.62 ± 0.08	0.94 ± 0.08	5.48 ± 0.20	5.02 ± 0.18	-11.19 ± 0.44	-96.52 ± 4.62

No significance compared with AAV-Vector group

Supplementary Table 4. mIPSC parameters in DG granule cells in the presence of pharmacological blockers. (related to Fig. 4g-i)

mIPSC	n	Frequency (Hz)	Rise 10-90% (ms)	Decay 90-37% (ms)	Half-width (ms)	Peak Amplitude (pA)	Area Under Curve (pAms)
WT	15	2.08 ± 0.23	0.62 ± 0.07	9.00 ± 0.53	7.80 ± 0.49	-30.71 ± 1.63	-426.23 ± 27.44
RIIβ$^{-/-}$	16	3.04 ± 0.27*	0.57 ± 0.04	7.68 ± 0.20*	6.86 ± 0.20	-29.68 ± 1.49	-369.38 ± 21.46

*$P < 0.05$ compared with WT group

Supplementary Table 5. mEPSC parameters in DG granule cells in the presence of pharmacological blockers. (related to Fig. 4j-l)

mEPSC	n	Frequency (Hz)	Rise 10-90% (ms)	Decay 90-37% (ms)	Half-width (ms)	Peak Amplitude (pA)	Area Under Curve (pAms)
WT	16	0.89 ± 0.11	1.32 ± 0.10	6.07 ± 0.18	6.15 ± 0.23	-11.68 ± 0.45	-109.13 ± 7.66
RIIβ$^{-/-}$	16	0.84 ± 0.08	1.04 ± 0.09	5.84 ± 0.33	5.55 ± 0.27	-11.56 ± 0.25	-101.83 ± 5.83

No significance compared with WT group

Supplementary Table 6. Electrophysiological properties of hippocampal DG granule cells at the normal RMP in the presence of pharmacological blockers. (related to Fig. 5)

Parameters	AAV-Vector (n = 13)	AAV-RIIβ-S112A (n = 12)
Input resistance (mΩ)	163.0 ± 10.3	200.3 ± 10.4*
RMP (mV)	76.3 ± 0.6	70.3 ± 1.1***
AP Threshold (mV)	-50.0 ± 1.3	-47.4 ± 1.4
AP Amplitude (mV)	113.9 ± 1.0	108.3 ± 1.5**
AP fAHP (mV)	11.3 ± 1.0	11.3 ± 0.9

*$P < 0.05$, **$P < 0.01$, ***$P < 0.001$ compared with AAV-Vector group

Supplementary Table 7. Electrophysiological properties of hippocampal DG granule cells at a fixed potential of -80 mV in the presence of pharmacological blockers. (related to Supplementary Fig. 3)

Parameters	AAV-Vector (n = 13)	AAV-RIIβ-S112A (n = 12)
Input resistance (mΩ)	145.1 ± 8.4	156.0 ± 6.9
AP Threshold (mV)	-50.3 ± 1.1	-49.1 ± 1.5
AP Amplitude (mV)	117.1 ± 1.0	115.5 ± 1.2
AP fAHP (mV)	10.8 ± 1.0	10.2 ± 0.8

No significance compared with AAV-Vector group

Supplementary Table 8. Electrophysiological properties of hippocampal DG granule cells at the normal RMP in the presence of pharmacological blockers. (related to Fig. 6)

Parameters	WT (n = 17)	$RII\beta^{-/-}$(n = 13)
Input resistance (mΩ)	188.2 ± 8.6	146.8 ± 9.6**
RMP (mV)	77.2 ± 0.7	80.3 ± 0.8**
AP Threshold (mV)	-50.4 ± 0.5	-49.5 ± 0.6
AP Amplitude (mV)	113.6 ± 1.1	113.2 ± 1.0
AP fAHP (mV)	7.4 ± 0.7	9.8 ± 0.6*

$^{*}P < 0.05$, $^{**}P < 0.01$ compared with WT group

Supplementary Table 9. Electrophysiological properties of hippocampal DG granule cells at a fixed potential of -80 mV in the presence of pharmacological blockers. (related to Supplementary Fig. 4)

Parameters	WT (n = 17)	$RII\beta^{-/-}$(n = 13)
Input resistance (mΩ)	174.0 ± 6.2	148.9 ± 7.9*
AP Threshold (mV)	-51.1 ± 0.6	-49.7 ± 0.7
AP Amplitude (mV)	116.5 ± 0.8	113.4 ± 1.0*
AP fAHP (mV)	7.8 ± 0.6	9.9 ± 0.5*

$^{*}P < 0.05$ compared with WT group

Supplementary Table 10. mIPSC parameters in CA1 pyramidal cells in the presence of pharmacological blockers.

mIPSC	n	Frequency (Hz)	Rise 10-90% (ms)	Decay 90-37% (ms)	Half-width (ms)	Peak Amplitude (pA)	Area Under Curve (pAms)
WT	12	5.45 ± 0.42	0.77 ± 0.03	11.07 ± 0.80	10.00 ± 0.70	-24.31 ± 1.41	-423.25 ± 34.84
RIIβ$^{-/-}$	14	6.13 ± 0.61	0.77 ± 0.08	8.68 ± 0.55*	7.70 ± 0.30**	-25.81 ± 0.86	-393.88 ± 21.49

$^{*}P < 0.05$, $^{**}P < 0.01$ compared with WT group

Supplementary Table 11. mEPSC parameters in CA1 pyramidal cells in the presence of pharmacological blockers.

mEPSC	n	Frequency (Hz)	Rise 10-90% (ms)	Decay 90-37% (ms)	Half-width (ms)	Peak Amplitude (pA)	Area Under Curve (pAms)
WT	10	0.77 ± 0.09	1.16 ± 0.08	6.02 ± 0.27	7.02 ± 0.31	-13.98 ± 0.35	-146.80 ± 10.37
RIIβ$^{-/-}$	8	0.54 ± 0.03*	1.66 ± 0.16**	5.97 ± 0.57	6.58 ± 0.67	-14.57 ± 0.60	-149.85 ± 9.48

$^{*}P < 0.05$, $^{**}P < 0.01$ compared with WT group
