# Supplementary material for: PKA-RIIβ autophosphorylation modulates PKA activity and seizure phenotypes in mice
Source: Commun Biol. 2021 Mar 1;4:263. doi: 10.1038/s42003-021-01748-4 (PMC7921646; doi:10.1038/s42003-021-01748-4)
Supplement: Supplementary file 3 — Description of Additional Supplementary Files [file 42003_2021_1748_MOESM3_ESM.pdf]

Description of Additional Supplementary Files

File name: Supplementary Data 1

Description: Source data underlying plots shown in figures and Supplementary tables.
