# Supplementary material for: PKA-RIIβ autophosphorylation modulates PKA activity and seizure phenotypes in mice
Source: Commun Biol. 2021 Mar 1;4:263. doi: 10.1038/s42003-021-01748-4 (PMC7921646; doi:10.1038/s42003-021-01748-4)
Supplement: Supplementary file 5 — Reporting Summary [file 42003_2021_1748_MOESM5_ESM.pdf]

nature research

Corresponding author(s): Zhuo Huang

Last updated by author(s): Dec 5, 2020

Reporting Summary

Nature Research wishes to improve the reproducibility of the work that we publish. This form provides structure for consistency and transparency in reporting. For further information on Nature Research policies, see our Editorial Policies and the Editorial Policy Checklist.

Statistics

For all statistical analyses, confirm that the following items are present in the figure legend, table legend, main text, or Methods section.

n/a	Confirmed	
☐	☒	The exact sample size (n) for each experimental group/condition, given as a discrete number and unit of measurement
☐	☒	A statement on whether measurements were taken from distinct samples or whether the same sample was measured repeatedly
☐	☒	The statistical test(s) used AND whether they are one- or two-sided<br>*Only common tests should be described solely by name; describe more complex techniques in the Methods section.*
☒	☐	A description of all covariates tested
☒	☐	A description of any assumptions or corrections, such as tests of normality and adjustment for multiple comparisons
☐	☒	A full description of the statistical parameters including central tendency (e.g. means) or other basic estimates (e.g. regression coefficient) AND variation (e.g. standard deviation) or associated estimates of uncertainty (e.g. confidence intervals)
☒	☐	For null hypothesis testing, the test statistic (e.g. F, t, r) with confidence intervals, effect sizes, degrees of freedom and P value noted<br>*Give P values as exact values whenever suitable.*
☒	☐	For Bayesian analysis, information on the choice of priors and Markov chain Monte Carlo settings
☒	☐	For hierarchical and complex designs, identification of the appropriate level for tests and full reporting of outcomes
☒	☐	Estimates of effect sizes (e.g. Cohen's d, Pearson's r), indicating how they were calculated

Our web collection on statistics for biologists contains articles on many of the points above.

Software and code

Policy information about availability of computer code

Data collection	All recordings were performed with a MultiClamp 700B amplifier (Molecular Device) and data were acquired using pClamp 10.6 software at a holding at normal MP or potential of -80 mV, filtered at 2 kHz and sampling rate at 33 kHz with a Digidata 1440A digitizer (Molecular Devices).
Data analysis	Ephys data were analyzed using pCLAMP 10.6 and MiniAnalysis software.

For manuscripts utilizing custom algorithms or software that are central to the research but not yet described in published literature, software must be made available to editors and reviewers. We strongly encourage code deposition in a community repository (e.g. GitHub). See the Nature Research guidelines for submitting code & software for further information.

Data

Policy information about availability of data

All manuscripts must include a data availability statement. This statement should provide the following information, where applicable:

- Accession codes, unique identifiers, or web links for publicly available datasets
- A list of figures that have associated raw data
- A description of any restrictions on data availability

The data that support the findings of this study are either available within the paper (and its Supplementary information files) or are available from the corresponding authors [ZH and RZ], upon reasonable request.

Field-specific reporting

Please select the one below that is the best fit for your research. If you are not sure, read the appropriate sections before making your selection.

☒ Life sciences ☐ Behavioural & social sciences ☐ Ecological, evolutionary & environmental sciences

For a reference copy of the document with all sections, see nature.com/documents/nr-reporting-summary-flat.pdf

Life sciences study design

All studies must disclose on these points even when the disclosure is negative.

Sample size	The sample size used in a study is usually determined based on the cost, time, or convenience of collecting the data, and the need for it to offer sufficient statistical power. Our sample size sufficiency was based on previous experiments from our laboratory.
Data exclusions	In the whole-cell configuration series resistance (Rs) 15-30 MΩ, and recordings with unstable Rs or a change of Rs > 20% were aborted.
Replication	All attempts at repetition or replication were successful.
Randomization	Mice were paired based on weight and age, and randomly selected for all experiments.
Blinding	The experiments were blind to viral treatment or drug treatment conditions during behavioral testing. The observer who evaluated neurological deficit scoring was blind to animal treatment. A investigator carried out electrophysiological recording without knowing the geonotype of mice before experiment. These tissue samples were assigned code numbers to blind. The analyses of electrophysiological data were performed by another independent investigator.

Reporting for specific materials, systems and methods

We require information from authors about some types of materials, experimental systems and methods used in many studies. Here, indicate whether each material, system or method listed is relevant to your study. If you are not sure if a list item applies to your research, read the appropriate section before selecting a response.

Materials & experimental systems

n/a	Involved in the study	
☐	☒	Antibodies
☒	☐	Eukaryotic cell lines
☒	☐	Palaeontology and archaeology
☐	☒	Animals and other organisms
☒	☐	Human research participants
☐	☒	Clinical data
☒	☐	Dual use research of concern

Methods

n/a	Involved in the study	
☒	☐	ChIP-seq
☒	☐	Flow cytometry
☒	☐	MRI-based neuroimaging

Antibodies

Antibodies used	Anti-PKA RIIβ (pS114) (BD 612550), Anti-PKA RIIβ (BD 610625), Anti-Phospho-CREB (Ser 133) (CST #9198), Anti-CREB (CST #9197), Anti-Phospho-PKA Substrate (CST #9624), Anti-GAPDH (Sigma G8795), Anti-β-actin (Sigma A5316)
Validation	The antibodies used in this study were commercial antibodies:<br>https://www.bdbiosciences.com/us/applications/research/b-cell-research/intracellular-antigens/human/purified-mouse-antipka-rii-ps114-47pka/p/612550;<br>https://www.bdbiosciences.com/us/applications/research/b-cell-research/intracellular-antigens/human/purified-mouse-antipka-rii-45/p/610625;<br>https://www.cellsignal.com/products/primary-antibodies/phospho-creb-ser133-87g3-rabbit-mab/9198;<br>https://www.cellsignal.com/products/primary-antibodies/creb-48h2-rabbit-mab/9197;<br>https://www.cellsignal.com/products/primary-antibodies/phospho-pka-substrate-rrxs-t-100g7e-rabbit-mab/9624<br>https://www.sigmaaldrich.com/catalog/product/sigma/g8795?lang=en&region=US;<br>https://www.sigmaaldrich.com/catalog/product/sigma/a5316?lang=en&region=US

Animals and other organisms

Policy information about studies involving animals; ARRIVE guidelines recommended for reporting animal research

Laboratory animals	Male C57BL/6 mice (18-20 g) of SPF grade were obtained from Vital River Laboratory Animal Technology Co, Ltd. (Permit number: SCXK 2012-0001). RIIβ knockout mice were provided by G.Stanley Mcknight (University of Washington).

Wild animals	This study did not involve wild animals samples.
Field-collected samples	This study did not involve field-collected samples.
Ethics oversight	We performed the experimental procedures in accordance with the National Institutes of Health Guide for the Care and Use of Laboratory Animals and the procedures were approved by the Biomedical Ethics Committee for animal use and protection of Peking University. Each effort was made to minimize animal suffering and the number of animals used.

Note that full information on the approval of the study protocol must also be provided in the manuscript.

Clinical data

Policy information about clinical studies

All manuscripts should comply with the ICMJE guidelines for publication of clinical research and a completed CONSORT checklist must be included with all submissions.

Clinical trial registration	2017PS045K
Study protocol	Patients (n = 355) with medically intractable TLE underwent phased presurgical assessment at Shengjing Hospital affiliated to China Medical University. Epilepsy diagnosis (including types and localization) as determined by clinical history, imaging examination (including MRI and/or PET), EEG (including the scalp and/or intracranial EEG), and psychological assessment. Patients with TLE caused by stroke, tumor, injury, and malformations were excluded from this study. In those selected for surgery, the hippocampus was resected according to standard procedures. Between July 2010, and February 2016, 246 hippocampi were resected. The study using clinical samples, which include 14 paired epileptogenic tissues and matched adjacent normal tissues, was approved by the Ethics Committee of Shengjing Hospital affiliated to China Medical University. Tissues were frozen in liquid nitrogen immediately after surgical removal and maintained at -80 °C until protein extraction. Informed consent was obtained from all subjects or their relatives.
Data collection	The Western Blot experiments from human tissues were performed and data were collected in Peking University
Outcomes	Tissues were used to perform the western blot analysis.
